# Creating innovation capabilities for improving global health: Inventing technology for neglected tropical diseases in Brazil

**DOI:** 10.1057/s42214-022-00143-y

**Published:** 2022-09-02

**Authors:** Paola Perez-Aleman, Tommaso Ferretti

**Affiliations:** 1 Desautels Faculty of Management, McGill University, Montreal, QC Canada; 2 Telfer School of Management, University of Ottawa, Ottawa, ON Canada

**Keywords:** emerging markets/countries/economies, innovation and R&D, networks, knowledge, policies, Sustainable Development Goals, capabilities and capability development, social innovation

## Abstract

Previous research on innovation capabilities in emerging economies shows knowledge networks tied to Western multinationals and national governments focused on economic growth. Less understood is the innovation capability building of emerging economies to achieve ‘good health’, an important Sustainable Development Goal. Here, we present a longitudinal study of a public research organization in an emerging economy and examine how it builds innovation capabilities for creating vaccines, drugs, and diagnostics for diseases primarily affecting the poor. We study FIOCRUZ in Brazil using archival, patent, and interview data about invention of technologies for neglected tropical diseases. We contribute novel insights into the evolution of knowledge networks, as national policy integrates innovation and health goals. We found significant diversification of local and foreign knowledge sources, and substantial creation of networks with public, private, and non-governmental organizations enabling collective invention. These R&D networks attract many multinationals to collaborate on socially driven innovation projects previously non-existent in their portfolios. The public research organization leads collaborations with multinationals and diverse partners, harnessing distributed international knowledge. Our results indicate emerging economies’ capabilities depend on elevating policies to increase health access for the poor to drive innovation and promoting local R&D to generate solutions to improve health.

## INTRODUCTION

Building innovation capabilities, or the organizational ability to create knowledge for new products and technologies, is essential for developing and emerging economies’ (DEEs) to confront gaps related to the Sustainable Development Goals (SDGs) (Mozas-Moral, Bernal-Jurado, Fernández-Uclés, & Medina-Viruel, 2020; Sachs, Schmidt-Traub, Mazzucato, Messner, Nakicenovic, and Rockstrom, 2019; UNCTAD, 2017). Efforts of DEEs to create knowledge are especially relevant for addressing SDG number 3: “Good Health and Well-Being,” which aligns with pressing social goals and national needs. Fulfilling this goal requires new health technologies for combating chronic infectious diseases, known as “neglected tropical diseases” (NTDs), that predominantly affect the poorest people (Hotez, 2020). NTDs constitute a global health problem that threatens over 1.5 billion people, accounts for an estimated 10 million deaths each year, and burdens economies with billions of dollars yearly of economic losses (Hotez, 2020; WHO, 2017). Historically, multinational companies (MNCs) lacked the commercial interest in addressing diseases affecting the poorest groups and neglected investments in research and development (R&D) to develop vaccines, drugs, and diagnostic tests against NTDs, which attracted only 1% of R&D investments for several decades (Moran, 2005; Morel et al., 2005; Muñoz, Visentin, Foray, & Gaulé, 2015; Pereira, Temouri, Patnaik, & Mellahi, 2013; Pereira et al., 2020; Trouiller, Olliaro, Rorreele, Orbinsky, Laing, & Ford, 2002). Recently, DEEs increased their innovation activities related to local problems connected directly with the SDGs (Montiel, Cuervo-Cazurra, Park, Antolin-Lopes, and Husted, 2021; UNCTAD, 2017). Understanding how DEEs build innovation capabilities to address major health challenges can inform how they acquire and create knowledge.

Recent research on innovation in DEEs analyzes knowledge networks and collaborations between public organizations and MNCs that develop new auto engines and parts, agricultural seeds, oil exploration technology, bioenergy enzymes, and software products (Corredoira & McDermott, 2014; Figueiredo, Larsen, & Hansen, 2020; Lema, Quadros, & Schmitz, 2015; Parente, Melo, Andrews, Kumaraswamy, & Vasconcelos, 2021; Perez-Aleman & Alves, 2017; Reynolds, Schneider, & Zylberberg, 2019). In Brazil, South Korea, and Taiwan, public–private knowledge networks contribute to building R&D capabilities in different sectors like electronics, automobiles, high-speed trains, agriculture, aircraft, and medicine (Amsden, 2001; Kim, 1997; Limoeiro & Schneider, 2019; McMahon & Thorsteindóttir, 2013; Parente et al., 2021; Rui & Bruyaka, 2021). Specifically, public research organizations (PROs) facilitate knowledge access for building production and innovation capabilities in DEEs across different industries (Lee & Yoon, 2015; Mazzoleni & Nelson, 2007; McDermott, Corredoira, & Kruse, 2009; Parente et al., 2021). The role of government institutions, particularly the PROs, is pivotal in addressing the innovation gap connected to local problems. However, few studies examine how the DEE PROs organize knowledge access and creation to facilitate innovation related to health challenges. Existing studies on NTD health challenges focus on global institutions and intellectual property rights (Vakili & McGahan, 2016). Others examine DEE pharmaceutical production capabilities (Horner, 2022). More general studies on innovation point to the important role of networks with connections across organizations with distributed knowledge (Figueiredo et al., 2020; Piore & Sabel, 1984; Powell, Koput, & Smith-Doerr, 1996; Saxenian, 2007). More research is needed for a deeper understanding of how DEEs organize for knowledge sourcing and innovation to address the health of the poor.

Existing studies show that PROs facilitate ties between national firms and MNCs, or participation in international networks led by MNCs, enabling external sourcing of local and international knowledge to produce new products (Lema et al., 2015a; McDermott & Corredoira, 2010; Perez-Aleman, 2011). Existing research also highlights that DEE PROs respond to local priorities (Mazzoleni & Nelson, 2007; Parente et al., 2021). In addition, research shows that DEE governments can steer industrial policies to generate market demand and production capabilities in competitive industries (Fuentes & Pipkin, 2019; Rodrik, 2007). Public policies also shape internal markets and partnerships to foster catch-up growth in areas responding to national welfare goals (Mazzucato, 2018). For example, public health policies in Brazil triggered industrial policies for local production to supply affordable and quality drugs (Flynn, 2008; Fonseca, 2018; Shadlen & Fonseca, 2013). The innovation gap in the NTDs means that inventions are needed. Globally, between 1975 and 1999, only 1% of new drugs were for NTDs (Trouiller et al., 2002). Between 2000 and 2011, only 1% of all clinical trials were for NTDs (Pedrique et al., 2013). The NTD health challenge requires fostering innovation in a context where MNCs neglect such investments to improve the health of the poor, and DEEs typically lack health R&D capabilities.

This study examines the case of FIOCRUZ, a Brazilian PRO, contributing to a better understanding of how DEEs acquire and create knowledge to build innovation capabilities for SDGs focused on healthcare challenges. We study the PRO role and its collaboration networks with MNCs and other organizations to access and generate the knowledge to develop NTD medical technologies. Our analysis uses original archival and patent data to capture the characteristics and evolution of knowledge networks that FIOCRUZ organizes with different domestic and foreign actors, including MNCs, that bring in varied knowledge for new NTD inventions.

The findings reveal DEEs build innovation capabilities by activating diverse knowledge networks that evolve in interaction with national social and innovation policies. Creating new technologies for NTDs depends on the PRO activating multiple ties with diverse organizations from the public, private, and non-governmental sectors, including local and international. Moreover, the PRO organizes connections with foreign MNCs in multiple forms, which are diverse in their geographic home, size, and age. In NTD innovation, foreign and domestic firms are actively integrated into the networks as vital knowledge generation partners, but PROs take the lead role in orchestrating knowledge sharing, recombination, and creation.

Social and innovation policies influence the shift towards innovation activities with higher degrees of complexity by enabling PROs to activate and enable networks for collective knowledge creation. The DEE PRO mobilizes and establishes local and cross-border collaborations to leverage local and foreign expertise to innovate novel products. Social policy drives DEE innovation efforts by creating demand for NTD health products. The DEE PRO aims to achieve innovation capabilities for improving the health status of poorer populations and reducing the social and economic burden that NTDs impose. Importantly, local actors focus on R&D for patenting, actively engaging in new knowledge creation to develop new NTD products. This study contributes an alternative model of DEE innovation capability building, offering a novel view of how a PRO, MNCs, and varied cross-sectoral organizations partner and interact in innovation networks to address major health challenges affecting the poor central for the SDGs.

## Literature Context: Innovation Capabilities and Knowledge Networks in Developing and Emerging Economies

Creating learning conditions to build innovation capabilities is central for DEEs as they proactively seek to innovate products and processes (Amsden, 2001; Anand, McDermott, Mudambi, & Narula, 2021; Lema & Lema, 2012; Malerba & Nelson, 2011; Perez-Aleman & Alves, 2017; Sabel, 1994). Learning and innovation depend on varied external know-how while combining knowledge to develop new capabilities (Nelson & Winter, 1982). Acquiring and creating knowledge for innovation relies on collaborations in the form of inter-organizational networks of different types of organizations (Figueiredo et al., 2020; Piore & Sabel, 1984; Powell et al., 1996; Saxenian, 2007). Interactive relationships through alliances and networks with diverse organizations, local and foreign, private and public, matter for building different knowledge and organizational capabilities (Anand et al., 2021; Hansen & Lema, 2019; McDermott et al., 2009; Parente et al., 2021; Perez-Aleman, 2011). Gaining expertise from a network of collaborators can bring various skills to generate new knowledge and develop innovation capabilities.

Some literature highlights the ties to MNCs as conduits making new knowledge available to DEE firms (Awate, Larsen, & Mudambi, 2015; Buckley, Strange, Timmer, & de Vries, 2020; Cano-Kollmann, Cantwell, Hannigan, Mudambi, & Song, 2016; Kumaraswamy, Mudambi, Saranga, & Tripathy, 2012; Lema et al., 2015a; Mudambi, 2008). In particular, existing studies highlight the knowledge DEE suppliers gain in networks with foreign MNCs in global value chains (GVCs) as they upgrade to higher value-added activities from production to innovation (Corredoira & McDermott, 2014; Ernst & Kim, 2002; Gereffi, Humphrey, & Sturgeon, 2005; Pietrobelli & Rabellotti, 2011). For example, Western MNCs in the automotive and information technology industries moved innovation activities related to their production operations to Brazil and India (Lema et al., 2015a). In these cases, the goals of Western MNCs drive collaborations between DEE suppliers and subsidiaries to create new products or processes closely connected to their production activities (De Marchi, Giuliani, & Rabellotti, 2018; Lema et al., 2015a; Mazzoleni & Nelson, 2007). In other instances, MNCs and DEE firms develop new technology together in dyadic relationships as strategic partners in user-producer interactions (Figueiredo et al., 2020). For example, Brazil’s Petrobras used a variety of networks to gain knowledge to create new offshore oil technologies for the global oil industry (Dantas & Bell, 2009). Similarly, Indian and Chinese firms used international collaborations to become leading producers in the photovoltaic, wind power, and electric vehicle industries (Lema & Lema, 2012). Increasingly, the ties between foreign and DEE firms are seen as a two-way interactive learning process (Herrigel, Wittke, & Voskamp, 2013).

As important, knowledge networks with government organizations are central to building innovation capabilities (McDermott et al., 2009; Parente et al., 2021; Perez-Aleman, 2005, 2011). Government institutions facilitate networks that enable DEE firms participating in MNC-led value chains to access and adapt different types of knowledge (Corredoira & McDermott, 2014). Ties and interactions with PROs and other governmental agencies increase knowledge flows to DEE firms to build capabilities (Kim & Nelson, 2000; Lundvall, 2010; Malerba & Nelson, 2011). PROs are privileged channels for knowledge contribution to local firms’ recombination (Laursen & Santangelo, 2017; Mazzoleni & Nelson, 2007; Mazzucato, 2018). Government agencies access foreign knowledge and support local innovation. Public–private networks usually feature in the DEE adoption of foreign technologies from advanced economies to achieve technological catch-up (Kim & Nelson, 2000).

Knowledge circulation and generation are prominent in networks with PROs that create conditions for building innovation capabilities in DEEs, such as Argentina, Brazil, South Korea, and Taiwan. For example, EMBRAPA, a Brazilian PRO, established an R&D network of regional and state-level institutes, private firms, and universities in the agricultural sector (Dahlman & Frischtak, 1993; Limoeiro & Schneider, 2019). In the soy seed business, EMBRAPA built innovation capabilities by creating new molecular biology and genomics techniques that attracted MNC partners (Parente et al., 2021). In the Argentinean auto sector, a PRO facilitated frontier knowledge from both local and foreign firms to advance supplier upgrading (Corredoira & McDermott, 2014). In South Korea, the Korean Institute of Electronics Technology and its Electronics and Telecommunications Research Institute promoted networks that benefited Samsung, Goldstar, and Hyundai during the 1980s (Kim, 1997; Mazzoleni & Nelson, 2007). In Taiwan, the Electronics Research and Services Organization conducted industrial research that promoted the formation of spin-off companies (Amsden, 2001). Likewise, Embraer, the Brazilian aircraft company, emerged as a spin-off from the Aeronautics Technology Center, a PRO that trained engineers and conducted R&D to build domestic technological research and design capabilities (Lee & Yoon, 2015).

Studies demonstrate that the government frequently creates pressures for innovation in DEEs, often connected to national priorities and local problem solving (Mazzoleni & Nelson, 2007; Parente et al., 2021; Pipkin & Fuentes, 2017; Stiglitz, Lin, & Patel, 2013). For example, overcoming dependence on oil imports drove Brazil’s initiatives to promote knowledge creation networks to innovate advanced biofuels from agricultural waste, attracting local and foreign firms (Perez-Aleman & Alves, 2017). The same import-substitution approach focused on industrial policy in the 1980s and 1990s fostered Petrobras’ buildup of frontier technological capabilities and leadership in international joint ventures (Dantas & Bell, 2009; Fuentes & Pipkin, 2019). Brazilian industrial policies in the automotive sector leveraged instead the subsidiaries and suppliers linked to leading Western MNCs to support the creation of flex-fuel engine technologies. This brought Brazil’s automotive suppliers close to the technological frontier (Fuentes & Pipkin, 2019). Similarly, government demand for health technologies that benefit the local population triggered networks to advance innovation in regenerative medicine with financial and social policy support (McMahon & Thorsteinsdóttir, 2013). PROs prominently drive these R&D networks (McMahon & Thorsteinsdóttir, 2013; Parente et al., 2021). These innovation efforts by DEEs that address local priorities engage public, private, and non-governmental organizations. They influence how MNCs might engage with the DEE markets and priorities. For instance, the MNC Sanofi-Genzyme established a Chagas disease research partnership with Brazil’s PRO FIOCRUZ in response to the Brazilian government’s goal to address local health problems, generating the corporation’s Social Responsibility Initiative (Bartlett, Khanna, & Choudhury, 2012).

While existing studies explore the growing innovation capabilities in DEEs, the majority focus on manufacturing, information technology, and agricultural sectors, giving less attention to healthcare. While advanced economies invest heavily in healthcare R&D, Western MNCs neglect R&D investments in diseases affecting the poorest in DEE countries (Muñoz et al., 2015). When DEEs face a high disease burden with limited innovation capabilities, whether and how they address the lack of inventions for locally relevant diseases influences access to healthcare. Improving human health is a Grand Challenge, meaning it encompasses important social problems of global impact (George, Howard-Grenville, Joshi, & Tihanyi, 2016). Health is also Goal 3 of the SDGs. Advancing innovation in drugs, vaccines, and diagnostics for NTDs is a Grand Challenge in healthcare (Vakili & McGahan, 2016). Existing studies primarily examine the development of production capabilities in DEEs, not how to create new technological knowledge (Ramani & Urias, 2018). As R&D is central to innovation that improves the health of the poor, there is a need for a deeper understanding of innovation capability building from the perspective of DEE organizations. The influence of national priorities in driving R&D and the role of the PROs matters for examining innovation capabilities in DEEs.

This study examines how PROs in DEEs promote and drive the creation of innovation capabilities targeting healthcare. Existing work on R&D knowledge networks in vaccines, drugs, and diagnostics highlights MNC leadership. What characterizes the networks PROs use to gain and generate knowledge for innovation? Given existing innovation capability gaps in DEEs, more analysis of DEE PRO organizations and their innovation-building efforts is needed. Specifically, how do government organizations facilitate DEE innovation capabilities to create NTD technologies? NTD innovation differs from well-studied experiences when Western MNCs lead new product development, as NTDs are not relevant for the MNC home markets. There is a need for studies on innovating new products that aim to improve the health of the poor from the perspective of DEEs. This study examines the process of building innovation capabilities targeting healthcare based on a case study of FIOCRUZ, a major player in health R&D and one of Brazil’s leading innovation organizations.

## Research Setting

The research setting to study how PROs in DEEs foster innovation capability building when addressing the SDG challenge of improving health is Brazil, one of the most innovative developing countries in healthcare (Vasconcellos, Fonseca e Fonseca, & Morel, 2018). Although the COVID-19 pandemic negatively affects its social progress, Brazil remains a model for improving health for the poor. From the mid-1980s to 2017, Brazil’s health policy goal was to make healthcare accessible to everyone (Fonseca, 2018; Shadlen & Fonseca, 2013). In the late 1990s and early 2000s, Brazil pioneered improved public health measures through accessible and affordable antiretroviral (ARV) drugs and social programs to fight against the AIDS epidemic, becoming a global model in this area, highlighting the importance of national policies to address health crises (Cassier & Correa, 2003; Flynn, 2008, 2014). After AIDS, Brazil addressed NTD challenges by directing efforts to build its innovation capabilities to create new health technologies (Cassier & Correa, 2019; Vasconcellos et al., 2018). Brazil’s push for NTD treatments, vaccines, and tests occurred as the government promoted innovation in its development strategy for two decades since the early 1990s (Perez-Aleman & Alves, 2017; Reynolds et al., 2019). Compared to other Latin American countries, Brazil has the highest average spending on R&D (as a percentage of GDP) in Latin America (Reynolds et al., 2019). Since the 1990s, Brazil continues to be among the few developing countries with growing strength in medical patenting and publications. In 2015, it ranked among the top 15 countries for innovative performance in health and R&D on NTDs, in terms of total medical patents filed by a country under the Patent Cooperation Treaty divided by its GDP per capita (Vasconcellos et al., 2018). Brazil’s government has targeted NTD healthcare issues since 1995. It was among the countries with the most scientific publications on NTD from 2005 to 2017 (Vasconcellos et al., 2018). Brazil also made significant progress in health biotechnology with public and private investments (McMahon & Thorsteinsdóttir, 2013).

Brazil sought to build production and innovation capabilities for the national goal of providing affordable access to health products that address basic needs and epidemics (Cassier & Correa, 2003; Ramani & Urias, 2018). Brazil’s 1988 Federal Constitution recognized universal free healthcare as a human right guaranteed by the national Unified Health System. The government aimed to increase access to primary health coverage for 120 million Brazilians, equal to 63% of the population (Andrade, Coelho, Xavier Neto, Carvalho, Atun, & Castro, 2018). In the late 1980s and early 1990s, the push to create universal access to healthcare generated an internal market demand for drugs and treatments that highlighted a mismatch with the pharmaceutical sector’s production capabilities (Shadlen & Fonseca, 2013). To address this discrepancy, the federal government pursued industrial policies promoting local production capabilities investing in public labs, and incentives for partnerships public–private and private–private (Fonseca, 2018; Ramani & Urias, 2018). Health assumed a strategic role in Brazil’s development agenda in the 1990s, viewed as central for economic growth, competitiveness, and innovation, given its links with different productive sectors and industries (Flynn, 2014). Brazil saw health as strategic for social inclusion and fostering innovation in the chemical, biotech, electronics, and services industries (Gadelha, Costa, Maldonado, Barbosa, & Vargas, 2013). Brazil’s drive to promote R&D on NTDs through the creation of national health institutes confronted the lack of interest from Western pharmaceutical MNCs, as these diseases were not relevant to their home country and did not offer a financial return (Barbeitas, 2019).

To examine how Brazil pursued innovation capability building, this case study’s focal actor is the Oswaldo Cruz Foundation, or FIOCRUZ, a PRO prominent for knowledge creation in the Brazilian health technology innovation system. Initially, the Brazilian government created FIOCRUZ as a public health science and technology organization to conduct research, invent and produce vaccines and drugs, and provide public health education and training (Delaporte, 2012). Established as a federal institute in 1907, it was first dedicated to eradicating yellow fever, bubonic plague, and smallpox epidemics when there was no local scientific health research in Brazil (Stepan, 1975). Modeled after the Pasteur Institute of France, it marked the beginning of Brazilian medical science research and practice, focused specifically on infectious and tropical diseases and developing serums and vaccines in response to public health needs (Delaporte, 2012). In 1970, it became a foundation, with a mandate to develop and manufacture drugs and vaccines while remaining part of the Health Ministry. It has over 12,000 employees (1000 with PhD degrees) and students in ten Brazilian states (Mazzucato & Penna, 2016). FIOCRUZ activities are consistent with Brazil’s efforts to reduce dependency on costly imported health technologies, build national health-related R&D capabilities, increase qualified human resources, and reduce inequality in access to healthcare (Fonseca, Shadlen, & Inacio Bastos, 2017). FIOCRUZ is central to Brazil’s innovation and government policies to build a health industrial complex that fulfills Brazil’s universal right to healthcare mandate (Gadelha et al., 2013). In 2012, the healthcare sector accounted for 25% of R&D spending in Brazil (Gadelha et al., 2013). The government features prominently in the country’s innovation efforts, with over half of R&D spending publicly funded and much of private R&D also state-funded (Limoeiro & Schneider, 2019).

Moreover, FIOCRUZ has a long history of research on NTDs, which cause diseases for 10 million Brazilians and 76 million people in Latin America (WHO, 2019). Since its inception, FIOCRUZ has been known for its work on Chagas disease, an NTD that accounts for 6000 annual deaths while affecting up to 4.6 million people in Brazil (Delaporte, 2012; Nobrega, de Araujo, & Vasconcelos, 2014). As NTDs are entrenched social issues in Brazil, policymakers pursued the build-up of new domestic capabilities. In 2014, FIOCRUZ received 25% of the total federal budget allocated to innovation agencies, the largest compared to other well-known research institutes in Brazil (Limoeiro & Schneider, 2019). FIOCRUZ appears prominently in international research networks and frequently publishes on NTDs (Fonseca e Fonseca, Sampaio, de Araujo, & Zicker, 2016).

## Methods

Between 2012 to 2018, we collected data on all NTD-related activities that FIOCRUZ conducted since its creation. We focused on all its R&D and production projects and noted all its relationships with other actors (public, private, plural, local, and international). The data collection included all its alliances and partnerships to capture what FIOCRUZ was learning from local, national, and global knowledge sources. We created an inventory of all the collaboration ties that FIOCRUZ established for NTD projects, including research, development, and production of therapeutics, vaccines, and diagnostics. The data collection included all NTD collaboration ties involving FIOCRUZ from its original founding in 1907, but mainly from its establishment as a foundation in 1970 until 2015, identifying 708 direct collaboration ties. In the inventory, three NTDs stood out as the target of FIOCRUZ’s collaborations due to their importance in Brazil: Chagas, malaria, and leishmaniasis. In Latin America, Brazil accounts for 25% of Chagas cases, 97% of leishmaniasis cases, and 36% of malaria cases (Hotez & Fujiwara, 2014; WHO, 2017).

Sources for building our FIOCRUZ partnership network inventory dataset include its Annual Activity Reports, in which FIOCRUZ describes its collaborations, and its partnership reports available on its website. As important, the Annual Reports of the Drugs for Neglected Diseases initiative (DNDI) provided information on partnerships and research consortia involving Brazilian organizations. To complement these sources, we also used corporate and organizational websites, newsletters, and annual reports of partner organizations and the collaborations that FIOCRUZ described in its reports. The data collected includes information on the organization and the nature of its partnerships (research consortia, strategic alliances, product development projects, technology transfer agreements).

Another data source was patent applications in Brazil from 1980 to 2015 to investigate trends in Brazilian R&D in the NTD technologies. We used the public international database Patentscope from the World Intellectual Property Organization (WIPO) to identify and count NTD-related invention applications filed at the WIPO Brazilian Patent Office and Brazil’s National Institute of Industrial Property (INPI). The Patentscope database (PCT) uses an abbreviated version of the IPC code that identifies and classifies each patent, which does not indicate the patent applicant’s origin. Therefore, we cross-referenced the patents found in PCT with the INPI and the European Patent Office’s Espacenet databases. This allowed us to obtain the two-digit country codes associated with each patent applicant that we retrieved in the Patentscope database. Additionally, we conducted a complementary search in the INPI and Espacenet to make sure that no relevant patent filed in Brazil was missing. As a result, we achieved a granular and comprehensive view of all the NTD-related patent applications filed in Brazil. Since inventions for patents filed in NTDs are difficult to classify by invention type due to the hundreds of different invention possibilities and compound descriptions, patents were extracted based on querying for associated disease names. The diseases chosen for the scope of the analysis include leishmaniasis, Chagas, tuberculosis, malaria, and HIV. Patents were queried in the various databases by disease name (both in English and Portuguese), scientific genus or disease species, and root word (if relevant).

To distinguish the different types of patent applications, we developed multiple categories building on existing literature on the NTD-related patent landscape (i.e., Pedrique et al., 2013; Vasconcellos & Morel, 2012) and the DNDi description of the pipeline for NTD-related product development (see, for example, DNDi, 2010). The categories are summarized in Table 1. They were applied by reading each patent document. From this patent data collection process, we produced a table with 570 entries specifying for each patent: IPC code, publication number, date applied/accepted, disease targeted, invention use, invention type, applicant(s), applicants’ country of origin, and a more detailed narrative description of the content of each patent. This output was crucial to studying the growth in Brazil’s innovation capabilities in the area of the NTDs and generating examples of innovations achieved by Brazilian organizations due to the expansion of FIOCRUZ’s international knowledge network. In particular, the patents’ origin, combined with their type, allowed us to develop a timeline of R&D activities, organizations, and areas of medical technology involved.

**Table 1:** Categories to distinguish and classify NTD-related patents filed in Brazil (1980–2015)

Category	Sub-category	Description
Diseases targeted	Chagas	Scientific genus “*Trypanosoma cruzi*”
	Leishmaniasis	Scientific genus “*Leishmania*”
	Malaria	Scientific name “*Plasmodium*”
	Tuberculosis	Scientific genus “*Mycobacterium*” was not used because shared with other different diseases. Only the species name was used
	HIV	Scientific genus “*Lentivirus*”
	Infectious diseases	Patents targeting a group of diseases not limited to a single NTDs, or patents targeting other relevant NTDs such as leprosy and dengue
Invention use	Diagnostic	An invention concerned with the medical diagnosis of an illness or disease or the diagnosis of a species, genus, or phenomenon
	Therapeutic	An invention concerned with the treatment, therapy and/or prevention of either an illness or disease
	Diagnostic and therapeutic	Inventions that fall in both categories
Invention type	Device	Any device with the intended use within the field of medicine for any medical purpose related to a given disease or illness
	Method	Any method or process or series of steps for performing a function or accomplishing a medical result
	Compound	A chemical substance composed of molecules or molecular entities formed of atoms from more than one element held together by a chemical bond
	Vaccine compound	A series of compounds for use in the creation of a product that stimulates a person’s immune system to produce immunity to a specific disease
	Drug compound	Any number of compounds for the creation of a product for medicinal use
	Topical medication	Any medication applied to a particular place on or in the body
Applicants’ origin	Brazil	The applicant is (the member of) a Brazilian organization
	Other	The applicant is from another country

In addition, a complementary source of data included 20 open-ended interviews with key informants at FIOCRUZ headquarters in Rio de Janeiro, including scientists and managers, during 2015 and 2016. These interviews with key actors in FIOCRUZ and Brazil working on NTD innovation focused on understanding their views on how they were building innovation capabilities and the resources they used to build them. Also, the interviews asked what activities were new and which changed over time. They were also asked to give specific examples of Brazilian NTD-related innovations and their experiences with partnerships and collaborations.

The combination of five decades of yearly reports, network development, patent applications, and interviews with key informants contributed to validating findings on both the evolution of FIOCRUZ, its R&D activities, partnerships, and knowledge sources to build NTD innovation capabilities in Brazil (see Table 2). This analysis draws on these three different sources, particularly the original inventory data of all NTD partnerships that FIOCRUZ established with other organizations from 1970 until 2015. Our database includes information on partner type (academic, research, private, public, NGO), partnership type, target disease for each project, and organization characteristics. Moreover, it includes detailed information about the goals of the relationship, NTD project focus, type of knowledge flowing through the social ties, innovation process, and chronology of partnership. We use software R for social network analysis to analyze data recorded in Microsoft Excel. For the data analysis, FIOCRUZ is the focal actor for the longitudinal analysis. This analysis emphasized chronological sequence, which revealed essential changes in patterns in the interactions and partnerships of FIOCRUZ’s networks that we use to analyze how it builds NTD-related innovation capabilities.

**Table 2:** Data summary

Data Source	Description	Analytical output
Network ties	708 direct NTD-related collaboration ties extracted based on an original inventory of all the collaborations established by FIOCRUZ with other organizations between 1970 and 2015	Longitudinal evolution of FIOCRUZ’s NTD partnership network characterized by partners’ type and partnerships’ nature and goals
Patent applications	570 NTD-related patent applications filed in Brazil between 1980 and 2015	Longitudinal characterization of the NTD patenting landscape in Brazil by type of applicant and invention
Interviews	Twenty (20) open-ended interviews with key informants at FIOCRUZ headquarters in Rio de Janeiro, conducted in 2015–2016	In-depth description of FIOCRUZ’ network development strategy to build innovation capabilities in the NTD area (1970–2015)

## Building NTD Innovation Capabilities: Fiocruz, Brazil (1970–2015)

**Figure 1:**
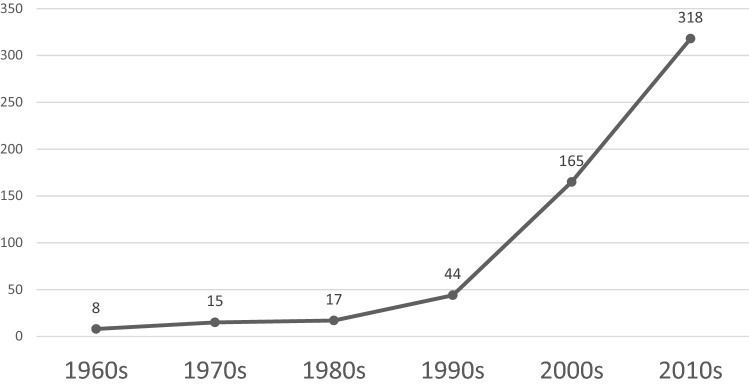
FIOCRUZ: evolution of NTD related network ties, 1960–2015. *Source* Authors’ elaboration from the original dataset.

Our findings indicate that since the 1980s, and particularly the late 1990s, Brazil has promoted the development and production of NTD therapies, vaccines, and diagnostics for nationally relevant diseases. Its strategy to build innovation capabilities for NTDs emphasized partnerships and consortia. FIOCRUZ established 17 NTD partnerships in the 1980s. This number doubled in the early 1990s to 40 and then increased six-fold to 109 by the early 2000s (Figure 1). This active creation of NTD partnerships and consortia continued in subsequent decades, reaching 318 inter-organizational collaborations by 2015 (Figure 1). Three patterns characterize the process of improving NTD innovation capabilities in FIOCRUZ and Brazil: first, actively creating collaboration networks with changing presence of local and foreign organizations and R&D orientation, including MNCs; second, collaborating with networks of increasingly diverse partners from the public, private and non-governmental sectors, and initiating patenting; and third, organizing collaborative ties for R&D and growth of patenting activity.

### 1970s–1980s: Building Networks with Mostly Local Organizations and International Partnerships for Technology Transfer

In the 1970s, FIOCRUZ became the Oswaldo Cruz Foundation, consolidating four state research centers and the graduate training program of the public health school into one organization. These research centers specialized in biological sciences, infectious diseases, maternal and child health, and endemic diseases. At the time, the Brazilian government identified weak local research capabilities, few Ph.D. level scientists, and scientific isolation (Azevedo, Ferreira, Petraglia, & Hamilton, 2002). Brazil established a National Immunization Program in 1973, and FIOCRUZ developed strong vaccine production capabilities. It created the Biomanguinhos Laboratory (Immunological Technology Institute) in 1976, following the country’s awareness of the importance of vaccines for preventing diseases. In addition, the public Farmanguinhos Laboratory (Medical and Drugs Technology Institute), dedicated to R&D in drugs and vaccines, merged into FIOCRUZ in 1976. Biomanguinhos became the largest producer of vaccines, test kits, and reagents to diagnose infections and parasitic diseases in Latin America (Oswaldo Cruz Foundation, 2012).

**Figure 2:**
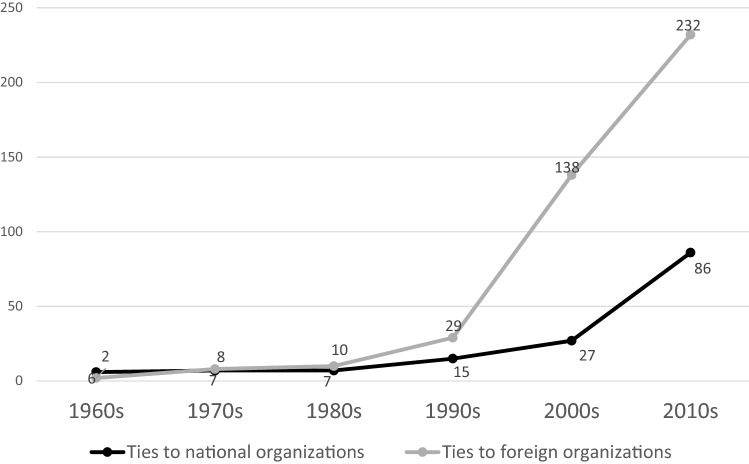
FIOCRUZ: National and International Partner Organizations in NTD related collaborations, 1960–2015. *Source* Authors’ elaboration from the original dataset.

In this period, FIOCRUZ increasingly developed partnerships; half were local organizations, primarily national research centers (Figure 2). In the 1980s, foreign organizations collaborating with FIOCRUZ included research centers and intergovernmental organizations. A third of all its collaborations focused on research (Table 3). Technology transfer collaborations accounted for another third of its partnerships (Table 3). Similarly, existing studies show collaborations between FIOCRUZ’s laboratories and foreign organizations, including research centers and MNCs, to transfer technology for local production for existing products to achieve social goals (Cassier & Correa, 2019; Guennif & Ramani, 2012).

**Table 3:** FIOCRUZ: Evolution of the innovation network for NTDs

	1960s	1970s	1980s	1990s	2000s	2010s
Total *N* of ties	8	15	17	44	165	318
Partnerships type	*Research collaborations* dominant (63%) *Technology transfer* relevant (25%)	*Research collaborations* prevalent (40%) *Technology transfers* prevalent (33%) *Financial support* relevant (20%)	*Research collaborations* prevalent (35%) *Technology transfers* prevalent (35%) *Financial support* relevant (18%)	*Research collaborations* dominant (59%) *Technology transfer* relevant (16%) *Financial support* marginal (7%)	*Research collaborations* dominant (67%) *Technology transfer* relevant (16%) *Technical collaboration* marginal (6%)	*Research collaboration* dominant (50%) *Capacity building* relevant (16%) *Policy development* marginal (11%) *Technology transfer* marginal (9%) *Technical collaborations* marginal (9%)
Partners type	*Research centers* dominant (75%)	*Research centers* dominant (53%) *Intergov. organizations* relevant (20%)	*Research centers* dominant (53%) *Intergov. organizations* relevant (18%) *Pharmaceutical MNCs* marginal (12% )	*Research centers* prevalent (41%) *Academic institutions* relevant (32%) *Pharmaceutical MNCs* marginal (14%) Intergov. organizations marginal (11%)	*Research centers* prevalent (38%) *Academic institutions* relevant (28%) Pharmaceutical MNCs relevant (18%) Intergov. organizations marginal (7%)	*Research centers* prevalent (30%) *Academic institutions* prevalent (30%) *Foreign governments* relevant (19%) *Pharmaceutical MNC*s marginal (13%)
Stages of the innovation process involved in the partnership	D*iscovery* dominant (67%) Development relevant (33%)	*Discovery* dominant (60%) *Development* relevant (27%) *Production* relevant (13%)	*Discovery* dominant (59%) *Development* relevant (29%) *Production* relevant (12%)	*Discovery* dominant (70%) *Development* relevant (20%)	*Discovery* prevalent (46%) *Development* relevant (29%)	*Discovery* prevalent (37%) D*evelopment* relevant (24%) *Access* relevant (16%)

Dominant: > 50% of the total ties, Prevalent: > 30% of the total ties, Relevant: > 15% of the total ties, Marginal: >5% of the total ties

*Source* Authors elaboration from the original dataset

Technology transfer collaborations with foreign companies contributed to building the capabilities of FIOCRUZ laboratories. During this period, FIOCRUZ prioritized improving its technological and production capabilities in vaccines for infectious diseases as demand increased, given the Brazilian government’s immunization goals. For example, FIOCRUZ established a partnership with Merieux Institute, a French company, in 1975 and another in 1978 to build its vaccine technology production capabilities. It also partnered with the Biken Institute of Osaka University in Japan, specializing in microbial diseases, to expand vaccine development and production. In 1982, Biomanguinhos had a technology transfer partnership with GlaxoSmithKline (GSK) to locally produce the oral polio vaccine, driven by Brazil’s effort to eradicate this disease. These alliances with Western MNCs and a foreign university were dyadic relations focused on achieving local production capability.

In 1978, FIOCRUZ developed diagnostic kits for Chagas and Leishmaniasis (Interview AB-B, 2015). Later in 1986, FIOCRUZ developed HIV test kits. Both diagnostic innovations were based on traditional test platforms. Since its founding, FIOCRUZ has focused on NTDs (Interview CC-FC, 2015). In 1909, the FIOCRUZ scientist Carlos Chagas discovered Chagas disease and its causative agent, the Trypanosoma Cruzi, its clinical manifestations, epidemiology, and entire life cycle (Delaporte, 2012). While FIOCRUZ advanced Chagas diagnostics, it could not develop drugs targeting the disease.

During this 1970–1980s period, FIOCRUZ had few relationships with pharmaceutical MNCs, which focused on technology transfer through bilateral relations (Table 2). For drugs, FIOCRUZ and its lab Farmanguinhos produced finished products from purchased imported inputs. At the time, there was no link between the industrial policy to produce locally existing drugs and the country’s innovation policy. FIOCRUZ did not rely on MNCs as the only foreign knowledge source and collaborated with a Japanese university with expertise in vaccine technology.

During this period, the activism of the Sanitarista Movement, Brazil’s most important health movement, influenced health policy orientation and implementation to focus on disadvantaged groups, too poor to buy medicines (Flynn, 2008; Gibson, 2017). The Movement had an important role in creating the 1988 Constitution that established the universal right to health. Its members, public health and medical scholars, and practitioners advocated for universality and equality in healthcare. At the same time, they also held positions in government organizations, including FIOCRUZ. These health activists eventually became directors of public health programs at the national and sub-national levels, promoting access to health for those traditionally excluded (Gibson, 2017). The health reforms promoted the engagement of FIOCRUZ labs, specifically Farmanguinhos in ARV production to confront the HIV/AIDS crisis (Flynn, 2008). By the end of this period, while promoting more access to vaccines and primary care, Brazil’s situation remained weak in terms of R&D, emphasizing industrial production. Nonetheless, the general transformation in Brazil’s health policy to improve health conditions for the poorest was connected to the efforts to build NTD innovation capabilities. The push for health reforms in the 1980s continued and reached new heights in the 1990s period, discussed next.

### 1990s–2000s: Increasing International Collaborations with Diverse Organizations while Initiating Patenting

In the 1990s, FIOCRUZ increasingly connected with Brazil’s national health policy goals of fighting endemic diseases, promoting access to medicines for low-income populations, and fostering innovation in health. There was a mutual shaping of the policy of the Brazilian Ministry of Health (MoH) and FIOCRUZ. As FIOCRUZ officials stated:

“Initially, we focused on the technology transfer approach. It was good for the economy and for industry, but it was limited for innovation” (Interview JC-FC, 2015).

“Since 2004, Brazil has been rethinking its innovation policies, and it created a new policy that included the Ministry of Health. The innovation policy was the result of public debate between FIOCRUZ, universities, the drug regulation agency, and the Ministry of Health” (Interview CC-FC 2015).

From the 1990s forward, FIOCRUZ formed networks with more international organizations, proactively initiating and joining local and global NTD initiatives to develop health technologies that were non-existent for most NTDs. Importing them was not an option. The underinvestment in global R&D related to NTDs created a lack of available therapeutic technologies, which limited Brazil’s ability to provide NTD diagnostics, drugs, and vaccines. FIOCRUZ moved to partnerships with MNCs that focused on co-development instead of transfer. FIOCRUZ also became a global leader in promoting NTD research and innovation. In addition, during this period, the variety of collaborators from different sectors increased, particularly from academic institutions.

There was a rise in collaborations for NTD projects between FIOCRUZ and international organizations. The number of collaborative ties tripled in the 1990s and quintupled in the 2000s (Figure 2). By the 2000s, its networks had evolved towards a predominance of international organizations, which accounted for 83% of its collaborators (Figure 2). At the same time, FIOCRUZ’s new partnerships increasingly focused on research and development. Research collaborations accounted for six out of ten partnerships in the 1990s and seven out of ten in the 2000s, and technology transfer alliances were no longer dominant as in earlier decades (Table 3). Research and the generation of new scientific knowledge constituted the foundation of the NTD efforts, more than developing production capabilities in connection to existing or re-engineered drugs. A FIOCRUZ official stated:

“We had all this scientific knowledge and yet no innovation of our own. This paradox pushed us into developing ways to overcome this major gap we have between our own research and the production of healthcare products. All we produced was from R&D done elsewhere outside of Brazil” (Interview MM-FP 2015).

Instead of technology transfer, FIOCRUZ pivoted to building more advanced biological research capabilities. For example, it established ties with research centers and academic institutions in the framework of the “Trypanosoma Cruzi Genome Initiative,” a project collaboration that started in 1994 to analyze the genomes of the parasite *Trypanosoma cruzi* (*T. cruzi*) involved in Chagas disease transmission to produce a low-resolution genome map and DNA sequence analysis. In Brazil, Chagas accounted for 77% of all deaths from NTDs in 2000–2011 (de Albuquerque, Dias, Vieira, Lima, & da Silva, 2017). In Latin America, around 6 million people are infected, with 70 million at risk of infection (WHO, 2017). The T. Cruzi Genome Consortium was a north–south network involving 20 labs, including Brazilian universities and those from Argentina, Venezuela, England, Spain, and the USA, plus the Pasteur Institute in France and the WHO (Degrave, de Miranda, Alex, Brandão, Aslett, & Vandeyar, 1997). Until the 1990s, developing countries were left out of major genome projects when involvement is crucial for developing drugs, vaccines, and diagnostics. In 1994, with the T. Cruzi Genome project, Brazil’s goal was to build advanced biological science capacity to support their scientific and technological development by participating in parasite genome sequencing and bioinformatics of infectious diseases affecting their population.

As important, increasing access to low-cost medicines became a cornerstone of Brazilian health policy during the 1990s, influencing the focus of international network development. FIOCRUZ became active in international campaigns advocating for access to essential medicines in 1999, a global health issue promoted by the NGO Doctors without Borders (Medecins Sans Frontieres, MSF). The relationship with MSF grew during these global campaigns. FIOCRUZ organized a new global network with MSF focused on NTD therapeutic innovation to promote access and availability of medicines for diseases affecting the poor. In 2003, FIOCRUZ co-founded the DNDI Working Group with MSF, which focused on new product development. This partnership also included public sector institutions from India, Malaysia, and Kenya and the United Nations Program for Research and Training in Tropical Diseases (UN-WHO-TDR). DNDI is now a non-profit R&D organization with more than 160 partners worldwide, focused on creating new NTD products using a patients’ needs-driven approach. FIOCRUZ brought its expertise when founding the DNDI network, enlisting the former executive director of Farmanguinhos in this effort (Cassier & Correa, 2019). A FIOCRUZ official recounts:

“We wanted to fill the gap between research and industrial activity. We needed different skills than basic research and production technology. The portfolio of FIOCRUZ was a majority technology transfer from foreign companies. This was a paradox given our scientific basis that we have no innovation produced by FIOCRUZ. Since the 2000s we have been developing ways to fill this gap” (Interview MM-FP 2015).

Brazil’s policy goal to create NTD technologies required investing in more discovery and development, as traditional import-substitution-oriented partnerships for drug development built on existing knowledge that was lacking in the NTD area. Consistently, FIOCRUZ actively engaged in developing new therapeutic and diagnostic technologies. Its first project focused on malaria, an infectious disease that ranked at the top of the NTD disease burden in Latin America, with 106 million people at risk (Hotez & Fujiwara, 2014). In 2002, FIOCRUZ developed a new therapy to treat malaria, the artesunate-mefloquine fixed-dose combination (ASMQ FDC) project, using improved technology and production of new anti-malaria drugs that required fewer pills and were less prone to resistance. FIOCRUZ conducted this project, known as the Fixed-Dose Artemisinin Combination Therapy (FACT) to treat malaria in Latin America and Asia, jointly with the DNDI network. FIOCRUZ, through the Farmanguinhos lab, led this consortium that involved French and Malaysian universities, start-ups, and private companies (Kameda, 2014). The French MNC Catalent contributed new recording methods. FIOCRUZ/Farmanguinhos became a scientific and industrial leader in this malaria-focused international network that involved pharmaceutical development and clinical trials. The novel technological knowledge developed at FIOCRUZ led to breakthrough technology. Achieving a chemically stable co-formulation for tropical conditions presented an enormous challenge (Davidson, 2006). Eventually, FIOCRUZ/Farmanguinhos developed the co-formulation of ASMQ into a single tablet (Davidson, 2006). It was a breakthrough in malaria treatment and the first Brazilian product collaborating with the DNDI network to be developed and registered in Brazil (DNDI, 2008). Subsequently, Brazil engaged in technology transfer following a south–south direction, as FIOCRUZ transferred the newly developed anti-malaria technology to CIPLA in India (Kameda, 2014). Notably, the rise of ties with organizations from other developing countries shows the FIOCRUZ strategy for NTD innovation fostered south–south collaboration dynamics.

During the 2000s, FIOCRUZ became particularly active in collaboration projects on Chagas disease, dengue, leishmaniasis, tuberculosis, and malaria, all with the purpose of innovating new products. FIOCRUZ networks predominantly focused on discovery and development in the innovation process, accounting for more than 70% of its partnerships (Table 3). These discovery activities generated and advanced scientific knowledge for novel treatments and diagnostics. Development activities included pre-clinical and clinical trials and testing to confirm the validity and feasibility of the new products. An example of a discovery activity was the Nitroimidazoles Proactive Compound Mining project, started in 2005 with DNDI, targeting Chagas and visceral leishmaniasis. A new drug was an objective as existing treatments were ineffective, required extended treatment periods, and with high toxicity. FIOCRUZ and its DNDI partners accomplished the discovery phase, including compound screening, lead selection, and lead optimization of antibacterial and antiprotozoal drugs to uncover new drug treatments. After identifying promising drug candidates, FIOCRUZ led the clinical development process to assess further development against *T. cruzi* and leishmania. Partners included organizations such as the Swiss Tropical Institute, Ouro Preto University from Brazil, and MNCs such as Covance and Biodynamics from the UK, and Sanofi from France.

A heterogeneous network of collaborative ties with diverse types of organizations from the public, private, and non-governmental sectors characterized the growth of FIOCRUZ partnerships (Table 3). Its partnership diversity included research centers, academic institutions, private companies, governments, and NGOs. While research centers accounted for nearly half of its partners until the mid-1990s, the relevance of academic institutions grew significantly since 1995, constituting a third of FIOCRUZ collaborators in this period (Table 3). At the national level, the ties with university partners intensified. This is consistent with studies showing that Brazilian universities increased their number of patents in the healthcare field during the 2000s (Cassier & Correa, 2019). Brazil’s prioritization of healthcare as a public policy goal fostered increasing efforts and resources for its public universities’ medical and biological departments in a coordinated effort to innovate pharmaceutical drugs and diagnostics for NTDs. The rise of collaborations between FIOCRUZ and Brazilian academic institutions is prominent in the 1990s, 2000s, and beyond (Table 3). This diversity of partner organizations, including governments, domestic and foreign private and non-governmental sectors, indicates that FIOCRUZ engaged in varied cross-sectoral knowledge sources to develop NTD innovations.

An increasing number of MNCs participated in projects with FIOCRUZ, focused on scientific research collaborations that constituted a significant share of the total partnerships that more than quadrupled in the 2000s and continued to increase into 2015 (Table 4). Foreign companies like GSK, Catalent, Biotools, Sanofi-Genzyme, and Chembio, among others, joined consortia and partnered with FIOCRUZ. Sanofi-Genzyme and FIOCRUZ partnered to work on new therapeutic treatments against Chagas disease (Bartlett et al., 2012). While at the beginning of this period, collaborations with six MNCs focused on tech transfer, by the 2000s, most of the growing number of partnerships with MNCs concentrated on joint research activities. When FIOCRUZ increased its collaborations with MNCs from six to 27, 56% were research collaborations compared to 37% technology transfers (Table 4). In addition, the new partnerships focused on the product discovery stage, absent in previous periods. In the 1990s, the ties with MNCs concentrated on product development and production. By contrast, in the 2000s, a third of the partnerships with MNCs focused on the discovery stage, and another half focused on product development (Table 4). Partnerships with GSK and Merck that previously focused on tech transfer shifted to engage in early research activities (Table 4). The new ties also widened FIOCRUZ’s product innovation with MNCs, including the discovery and development of drugs and diagnostics for NTDs such as Chagas and leishmaniasis.

**Table 4:** FIOCRUZ: evolution of NTD partnerships with MNCs

	1970s	1980s	1990s	2000s	2010s
Total N# ties with MNCs	1	2	6	27	38
Partnerships type (% of the total ties)	Technology transfer only	Technology transfer only	Technology transfer only	Research collaboration (56%) Technology transfer (36%) Patent Development (4%) Capacity Building (4%)	Research collaborations (57%) Technology transfer (34%) Patent Development (3%) Capacity Building (6%)
Stage of the innovation process addressed in the partnership (% of the total ties)	Development only	Development only	Development only	Development (52%) Discovery (37%) Testing (11%)	Development (47%) Discovery (34%) Testing (13%) Production (6%)
Partnership structure (% of the total ties)	Bilateral only	Bilateral only	Bilateral only	Bilateral (52%) Multilateral (48%)	Bilateral (50%) Multilateral (50%)

*Source* Authors elaboration from the original dataset

Significantly, during the 2000s period, consortia grew in importance and focused on creating new pharmaceutical and diagnostic technologies. FIOCRUZ collaborated with MNCs in projects involving multiple and diverse partners, not just bilateral ties as in the previous decades (Table 4). A large part of the newly established research and discovery ties involved multilateral partnerships or consortia, where the MNCs collaborated with FIOCRUZ and other research and non-governmental sector institutions. Sometimes, multilateral projects involved multiple MNCs and local companies, as well as government labs and universities. For example, in 2002, FIOCRUZ’s FACT Project involved a partnership that included the French MNC Catalent and five other organizations from the public and non-governmental sectors. Similarly, the Nitroimidazoles Proactive Compound Mining Project to discover new compounds for creating Chagas and other NTD-related drugs involved a partnership between FIOCRUZ and 18 organizations, including eight MNCs, such as Covance (UK), TB Alliance and Sigma-Aldrich (USA), and Sanofi-Aventis (France). The majority of MNCs partnering with FIOCRUZ were from Western countries. In this period, the FIOCRUZ strategy promoted participation in emerging multilateral projects involving MNCs for research collaborations geared towards product development or discovery.

In this post-1990s period, FIOCRUZ also promoted the creation of innovation capabilities in diagnostics, such as blood tests. MNCs dominated the global blood test market (Kameda, 2019). Entry barriers were high given the requirements for complex molecular biology and automation skills. Facing high international prices while establishing a policy for mandatory public blood-screening routines, Brazil’s MoH stimulated the local production of tests in 2002. In 2005, FIOCRUZ/Biomanguinhos organized a consortium that included biologists from the Federal University of Rio de Janeiro (FURJ), the IBMP, the Brazilian company HEMOBRAS, and foreign MNCs such as Qiagen from Germany. This product development consortium invented diagnostic tools to screen relevant infectious diseases in Brazil and increase local access to other biological tests and safe blood products. The consortium successfully met the national demand for nucleic acid tests (NATs) and supplied the diagnostic inputs from 2009 onwards (Bonfim, Goncalves, Moreire, & Jacometti, 2016). FIOCRUZ/Biomanguinhos invented the molecular biology components of the control module that calibrates kit reactions, as well as a 2010 patent application registered in Brazil (Kameda, 2019). The MNC Qiagen transferred one of the extraction modules used in the new NAT test. This global product development partnership illustrates how FIOCRUZ used multilateral ties linking together government labs, local universities, and private companies to source and create knowledge.

The increased number of consortia where foreign MNCs form part of multilateral networks focused on R&D, while bilateral alliances for technology transfer continued. Interestingly, these alliances leveraged existing knowledge to create innovation rather than produce the same product. For example, a partnership between FIOCRUZ and the North American Chembio Diagnostics company targeted innovating diagnostic tests in 2003, when FIOCRUZ-Biomanguinhos had a technology transfer agreement for producing HIV quick diagnostic tests (Interview AB-B, 2015). This partnership focused on acquiring and building knowledge for rapid diagnostic test platforms, which helped FIOCRUZ move to innovate a biotech platform beyond their traditional test platform developed and produced for Chagas and leishmaniasis in the 1970s (Interview AB-B, 2015).

**Figure 3:**
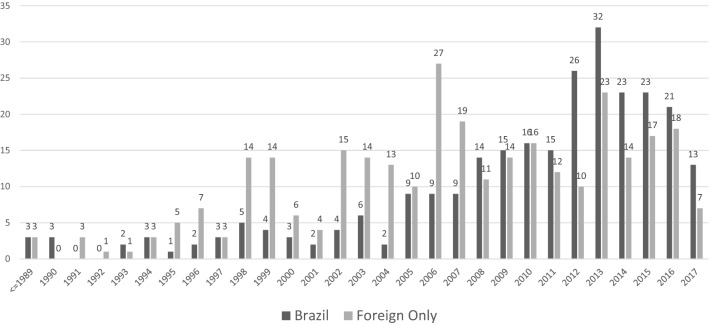
NTD patent applications over time: Brazil involved vs. foreign applicant only (1989–2017). *Source* Authors’ elaboration from the original dataset.

There was an increase in patenting in the late 1990s and post-2000s (Figure 3). In compliance with the 1995 World Trade Organization TRIPS agreement, Brazil began to grant patents in biotechnology and pharmaceuticals in 1997, facilitated by the 1996 national patent laws reform (Ryan, 2010; Shadlen, 2009). Subsequently, the Innovation Law passed in 2004 was designed to support riskier research investments and facilitate technological transfers from academic and research sectors to private (Dos Santos & Torkomian, 2013). Of the patents applied for since 1990, 47% of NTD-related patents filed in Brazil are of Brazilian origin. The significant increase in applications involving Brazilian institutions highlights the building of local R&D capabilities. The number of applications involving Brazilians grew significantly and now exceeds foreign applications. Until 2007, foreign-only NTD patent applications exceeded those with Brazilian applicants. Post-2008, the number of Brazilian applications consistently exceeded foreign-only applications (Figure 3).

The growing number of public and private universities partnering with FIOCRUZ also coincided with their higher share of patent applications (48%), followed by biotechnology and pharmaceutical companies (28%) and research centers (non-government and non-university) (26%). Combined, Brazilian academic and research institutions represent 74% of Brazilian patent applications for leishmaniasis, Chagas, malaria, and tuberculosis from the mid-1990s to 2017. The relevance of academic institutions shows in their significant share among FIOCRUZ partners in national and international networks; after 1995, they accounted for a third of its total partners (Table 3) and up to 40% of its Brazilian partners. This is consistent with findings on the pharmaceutical sector in Brazil that indicate universities and research institutions increased innovative activities in producing new technology. While from 2000 to 2005, universities and public research institutions represented only 3.1% of patent applications filed by Brazilians, they comprised over 60% of applications filed in the organic chemistry and biotechnology fields, and from 2007 to 2011, university patent applications grew by 450% compared to 2005 (Caliari, Mazzoleni, & Costa Póvoa, 2013).

### 2010s–2020: Growing Patenting Activity, R&D with MNCs, and Diverse International Ties

During the last decade, FIOCRUZ further expanded its network of collaborators, focusing on ties to varied international organizations and increasing patenting efforts and research and product discovery in partnership with MNCs. These actions build on FIOCRUZ’s leadership in promoting NTD initiatives in Brazil and as part of the DNDI network. For example, FIOCRUZ finalized an agreement with the Pernambuco State Pharmaceutical Laboratory (LAFEPE) in Brazil that delivered the first pediatric formulation of benznidazole, the most widely used drug for the treatment of Chagas disease. This partnership created an affordable, easy-to-use drug for worldwide distribution. It was an important achievement as 9000 children globally are born with Chagas every year (WHO, 2017). In 2010, of the 8 million affected worldwide, 14,000 children were in South America (DNDI, 2010). Until 2008, only adult treatments were available. Eventually, the collaboration between FIOCRUZ and LAFEPE led to a new pediatric treatment registered in Brazil in 2011. In 2013, the World Health Organization (WHO) included this new Chagas treatment as an essential medicine. In 2017, the product was registered in the US and other Latin American countries. It was the first drug approved by the Food and Drug Administration to treat Chagas in the US, where more than 300,000 people are affected (DNDI, 2018). Ongoing R&D for discovering new compound leads for Chagas and leishmaniasis treatments continues with the Lead Optimization Latin America Project (LOLA), which involves collaborations with DNDI, the Universities of Sao Paulo and Campinas, the WHO/TDR, and pharmaceutical companies.

It is novel for Brazil to move into early drug development activities and identify new compounds to develop NTD therapies. Previous efforts to implement the federal government’s health policy goals had first focused in the 1980s and early 1990s on developing capabilities related to existing compounds and products. Since the mid-1990s, Brazil moved to more R&D-intensive partnerships for product development. In this latest phase, the health industry complex shows an increased ability to drive the entire R&D process for drugs and therapies. In fact, Brazilian NTD-related patents increased post-2000 (Figure 3), and the patenting of compounds accounted for 70% of them. Moreover, a sample of NTD inventions from 1980 to 2017 demonstrates that FIOCRUZ was active in patenting compounds for the treatment, prevention, and diagnosis targeting multiple infectious diseases (Table 5).

**Table 5:** Selected examples of NTD inventions patented by FIOCRUZ, Brazil

Disease targeted	Invention use	Invention type	Patent description	Date published
Chagas	Diagnostic	Compounds	Antigens and synthetic polypeptides for use in diagnosing Chagas	1991
Chagas	Therapeutic	Compounds	Synergistic antagonists used in combined therapy with prio-oxidizing drugs, specifically benznidazole	2010
Chagas	Therapeutic	Compounds	Pharmaceutical compositions for detecting and controlling tricatoms, the vectors of Chagas disease	2005
Chagas	Therapeutic	Compounds	*Lentinus strigosus* extracts and pharmaceutical compositions used for the inhibition of cancer tumors and Chagas disease	2008
Chagas	Diagnostic	Method and compounds	Method kit for the detection of *T. cruzi* in saliva	2001
Infectious diseases	Diagnostic	Method and compounds	Process of preparing antigen compositions for the use in diagnosing infectious diseases	1992
Infectious diseases	Therapeutic	Compounds	Compounds for treatment or prevention of infectious diseases	2008
Leishmaniasis	Therapeutic	Compounds	Polypeptides for use in the treatment of leishmaniasis	2006
Leishmaniasis	Diagnostic	Compounds	Use of recombinant leishmania antigens in detecting, identifying, and qualifying specific antibodies in biological material to be used for the diagnosis of leishmaniasis and further uses (treatment, vaccines)	2010
Leishmaniasis	Therapeutic	Compounds	Vinca alkaloid extracts used for treating leishmaniasis	2000
Leishmaniasis	Therapeutic	Compounds	Metabolites and inhibitors of leishmaniasis	2009
Leishmaniasis	Therapeutic	Compounds	Process of obtaining compounds and derivatives for the treatment of leishmaniasis	2008
Leishmaniasis	Diagnostic therapeutic	Method	Process for preparing antigens for diagnosis of and vaccination for cutaneous and visceral leishmaniasis	1990
Leishmaniasis	Therapeutic	Compounds	Pharmaceutical composition containing alagoa red propolis extract in combination with injectable meglumine antimoniate for treating tetanus leishmaniasis	2017
Leishmaniasis	Diagnostic	Method and compounds	Molecular differentiation of leishmania species	2002
Malaria	Diagnostic	Compounds	*Plasmodium gallinaceum* antigens for use in the diagnosis of malaria	2001
Malaria	Therapeutic	Compounds	Antimalarial composition using a combination of natural polyphenol curcumin and sodium diethyldithiocarbamate for the treatment of malaria	2018
Malaria	Therapeutic	Compounds	Compounds derived from artesunate for the treatment of parasitic diseases such as malaria	2005
Tuberculosis	Diagnostic	Vaccine compounds	Recombinant. Mycobacterium proteins for the diagnosis of tuberculosis and a tuberculosis vaccine	2015
Tuberculosis	Therapeutic	Compounds	Pharmaceutical composition for the treatment of tuberculosis	2017
Tuberculosis	Therapeutic	Compounds	A-ketoacyl compounds used to develop new derivatives of isoniazid for the treatment of tuberculosis	2016
Tuberculosis	Therapeutic	Compounds	Azole compounds used as tuberculostatic and leishmanicidal agents and pharma compositions	2007

*Source* Authors elaboration from the original dataset

Yet another example of development activity is the PodiTrodi project (2011–2017), a Brazilian–European cooperation project to produce a new diagnostic platform for Chagas and other tropical diseases. This new product development effort produced a device that integrates microsystems (novel biosensors and microfluidics) and control electronics suitable for point-of-care diagnosis to detect Chagas disease. The new technology included four areas of research (bioassay; the active microfluidic cartridge; the novel biosensor; and the instrumentation) done by this consortium of five Brazilian labs and five European labs (Italy, France, Germany, Portugal, and Finland), jointly funded by the European Union, the Brazilian Council of Scientific and Technological Development (CNPQ), and the Brazilian National Development Bank (BNDES). For example, in 2015 and 2016, BNDES granted non-refundable loans to develop new therapeutic and diagnostic technologies for Chagas, Leishmaniasis, and Tuberculosis, led by FIOCRUZ in partnership with DNDI (Cassier & Correa, 2019).

From 2010 forward, FIOCRUZ established partnerships with universities in Brazil to develop nucleic acid tests (NATs) in blood screening technologies. A consortium between FIOCRUZ/Biomanguinhos laboratory and the Brazilian Molecular Biology Institute of Parana (IBMP) contributed to developing NAT diagnostics for Zika, dengue, and Chikungunya in 2016 (Kameda, 2019). These are mosquito-transmitted diseases associated with poorer living conditions; for example, in 2014, Brazil ranked at the top in Latin America with 21 million cases of dengue disease, accounting for 40% of the Latin American burden for this NTD (Hotez & Fujiwara, 2014). The FIOCRUZ collaboration model of working in consortia built on its previous experiences working with Brazilian universities to develop blood tests for infectious diseases, such as HIV and hepatitis.

At the same time, the Brazilian MOH commissioned projects to identify better and new treatments for visceral leishmaniasis (VL). Brazil accounts for 97% of the VL disease burden in Latin America, prevalent among the poor (WHO, 2020). In 2012, FIOCRUZ-Belo Horizonte organized a consortium of five Brazilian universities, a local hospital, and DNDI (Barbeitas, 2019). FIOCRUZ and its partnership with DNDI stimulated R&D for new product development. Developing new treatments for leishmaniasis was a priority for Brazil as existing drugs were outdated, expensive, highly toxic, difficult to administer, and non-specific (Barbeitas, 2019). This cross-sectoral R&D collaboration between FIOCRUZ, universities, and international NGOs created new therapeutic strategies combining diverse scientific, industrial, and organizational expertise. Another collaboration between FIOCRUZ and Brazilian universities developed a vaccine to prevent cutaneous and Visceral Leishmaniasis, which led to a patent filed in 2016.

A FIOCRUZ official noted an innovative accomplishment:

“One cutting edge example of our effort to innovate is in Schistosomiasis. The WHO selected the vaccine made in FIOCRUZ, which entered the second phase of clinical trials in 2015. It is the first fully developed Brazilian vaccine, the only parasitic vaccine in the world against Schistosomiasis and Fasciolosis” (Interview JC-FC 2015).

Post-2010, FIOCRUZ increasingly fostered local and international collaborations for capacity building and policy activities; between 2011 and 2015, these accounted for nearly 30% of its partnerships (Table 3). FIOCRUZ helped create capabilities in public and non-governmental organizations in African and Latin American countries as part of the south–south collaborations that characterize innovation efforts in the NTD field. FIOCRUZ continued with its characteristic local and global outlook, like the FACT Project that transferred malaria therapeutic technology from Brazil to India. For example, FIOCRUZ supported the Mozambique National Institute of Health to build technical capacity among their local public health workers in 2012. On the policy side, FIOCRUZ supported the Network of Public Health Institutes of Portuguese-speaking countries by developing evidence-based public health policies and supporting scientific knowledge sharing as part of efforts to develop and implement policies to combat NTDs. As important, FIOCRUZ worked to build the capacity in other regions of Brazil, reaching beyond its headquarters in Rio de Janeiro, particularly strengthening labs in the north, south, and Amazon regions of the country, contributing to the rise of ties with domestic partners in the 2015 period (Figure 2).

In this period, the FIOCRUZ strategy further expanded partnerships with MNCs and intensified the research collaborations. In the 2010–2015 period, FIOCRUZ established 38 ties with MNCs, 57% of which were research collaboration partnerships to jointly create new knowledge, and only a third focused on existing technology transfer (Table 4). Continuing as in the previous decade, half of all FIOCRUZ partnerships with MNCs were in multilateral consortia involving joint efforts with other organizations, particularly at the discovery and development phase of product innovation (Table 4). FIOCRUZ collaborated with MNCs, such as the A-ParaDDise research collaboration consortium launched in 2011 involving 12 organizations, including Swedish MNCs Kancera and Adlego Biomedical. In this joint effort, FIOCRUZ, MNCs, and the other research organizations identified enzymes crucial for the survival of the parasites *Schistosoma mansoni*, *Trypanosoma cruzi*, *Leishmania braziliensis*, and *Plasmodium*, which would constitute the target of future drugs to fight the NTDs in Brazil and globally.

Another example is the FIOCRUZ consortium project Podi-Trodi, which partnered with the MNCs ST Microelectronics (Italy), Haecker Automation (Germany), and BiFlow Systems (Germany) and nine more organizations from academia, the public sector, and civil society. Podi-Trodi created a new portable technology supporting point-of-care diagnostic for NTDs in remote areas. FIOCRUZ promoted and relied on multilateral partnerships involving MNCs to build innovation capabilities for new product discovery and development.

At the same time, while FIOCRUZ emphasized more R&D partnerships with MNCs in research-focused consortiums, it continued to establish new bilateral partnerships for technology transfer and research. For example, FIOCRUZ partnered with Japanese MNC Eisal Co. to generate new knowledge to develop drugs for malaria and NTDs. Important partnerships continue seeking an increase in production capabilities and enabling product development to satisfy the state-led demand for NTD drugs. For instance, FIOCRUZ partnered with MNCs from other DEEs, such as the pharmaceutical MNC Lupin from India, with whom FIOCRUZ developed the capability to manufacture Tacrolimus capsules with the support of the MoH and the WHO. New bilateral collaborations also were outgoing. For example, FIOCRUZ transferred NTD-related technology to Cuban firms Herber Biotec and Cimab. Today, FIOCRUZ is the central actor in networks with MNC partners from the West and the South, as a recipient of more advanced technology, a provider of frontier technology, and a counterpart for mutual learning and knowledge creation in the area of NTDs. Markedly, FIOCRUZ changed its approach with MNCs from agreements that emphasized tech transfer of existing products to co-development of new therapeutics and diagnostics (B. Interview, 2015).

Brazilian patenting growth continued in the 2010–2017 period, with patent applications from national applicants consistently surpassing foreign only ones since 2011. In terms of technology, therapeutic-related patents constituted three-quarters, and diagnostics products a quarter (see examples in Table 5). Patents related to leishmaniasis accounted for a third of the patents, followed by tuberculosis, malaria, and Chagas. The important presence of academic institutions as patent co-applicants remained constant, accounting for half of the patent applicants. After academic institutions, biotech and private pharmaceutical companies were relevant partners. For example, GSK applied for patents in Brazil. However, MNCs were not the only or main actors innovating, as local universities and FIOCRUZ had an active role as innovators in NTD therapeutics and diagnostics. The growing patenting in this period and the use of bilateral and multilateral partnerships that included MNCs indicate a decisive shift from seeking technology transfer to co-development with diverse partners.

The new global networks contributed to expanding Brazil’s capabilities and allowed FIOCRUZ and local organizations to draw on expertise and knowledge sharing from different countries and organizations from the public, private, and non-governmental sectors. FIOCRUZ also supported innovation through national resourcing of knowledge and government financing while bringing in foreign scientific and technical expertise. This means a strategy to build innovation capabilities with less dependence on MNCs. At the same time, FIOCRUZ/Biomanguinhos became the largest production center for vaccines, kits, and reagents of infectious diseases in Latin America (Oswaldo Cruz Foundation, 2011).

## Discussion: Knowledge Creation Networks for DEE Innovation Capability Building Interact with Social Policy Goals

### Alternative Knowledge Creation Networks for Social Goals, not Led by MNCs

This study contributes a novel view of the process of building innovation capabilities in DEEs. Knowledge flows crucial for building innovation capabilities depend on interactive learning in collaborative networks involving diverse organizations (Cano-Kollmann et al., 2016; Piore & Sabel, 1984; Powell et al., 1996). In the literature focusing on DEEs, these networks are associated with cross-border knowledge flows in the context of GVCs and MNCs. MNCs are seen as the crucial link between innovation in advanced and developing economies, with PROs facilitating knowledge access and recombination (Anand et al., 2021; Figueiredo et al., 2020; Lema et al., 2015a; Saxenian, 2007). Consistent with that line of research, this study also finds that international knowledge networks matter for building DEE innovation capabilities. However, we provide a novel view of these networks, highlighting a changing role for the MNC and the PRO that evolves with DEE innovation and social policies. While MNCs remain relevant, the knowledge sources crucial to creating new technology become highly diversified as DEE governments set increasingly ambitious innovation goals. Moreover, the PRO actively leads the creation, expansion, and diversification of knowledge networks driven by national policy goals that influence the MNC’s engagement with innovation activities in DEEs. In this sense, the policy context matters for facilitating the emergence and growth of networks for cross-border and local knowledge flows for innovation in DEEs. Importantly, the policies involved are socially driven, different from those receiving the most attention in the literature, such as production and trade policies.

Networks that facilitate international knowledge flows for building innovation capabilities in DEEs are not automatic or given by foreign actors. Existing work emphasizes cross-border networks in which Western MNCs are the main knowledge conduits, often in GVC production linkages with DEE suppliers (Corredoira & McDermott, 2014; Figueiredo & Cohen, 2019; Herrigel et al., 2013; Lema et al., 2015a). This study advances our understanding of innovation-focused networks by showing that the DEE PRO actively organizes collaborations creating alternative paths to gain global knowledge. The MNCs join networks with diverse organizations centered on R&D projects that the foreign company did not already conduct at home or in the DEE host country. The R&D networks also focus on products that the MNC did not produce previously at home or in the DEE due to lack of commercial interest. As the DEE PRO assembles knowledge of different types to facilitate the development of DEE innovation capabilities, it does not depend or rely on established GVC linkages or pre-existing partnerships with MNCs. The DEE socially driven innovation goals promote the PRO’s active organizing of the emergent R&D networks.

This study elucidates the evolution of the characteristics of networks for knowledge access and generation in a context that initially lacks innovation capabilities and entrepreneurial demand for innovation. Existing studies show that networks evolve in contexts where innovation capabilities are well developed (Powell et al., 1996; Powell & Grodal, 2005). In addition, partnerships and private investments in R&D activities emphasize profitability (Rodrik, 2007). Our study contributes new insights on knowledge networks evolution in contexts lacking such capabilities and commercial interest, advancing initial work showing an interaction between existing organizational capabilities and network evolution in DEEs (Dantas & Bell, 2009). When conducting little R&D, the networks historically focused on building production capabilities related to existing products developed abroad by foreign MNCs. These ties arise in response to the DEE government’s goal of locally producing traditionally imported medicines. Existing studies show that importing finished products or active ingredients is the typical commercial relationship for DEEs in the pharmaceutical industry (Horner, 2022). As the DEE goal shifts from production to increasing innovation capabilities, an active intent to create R&D-intensive networks emerges to develop new vaccines, drugs, and tests for NTDs. Instead of relying on partnerships with one MNC partner, the DEE R&D networks comprise multiple foreign and local partners, including diverse MNCs, private firms, universities, and non-governmental organizations. Both the local and foreign knowledge collaborations increase, thereby growing multidirectional learning ties.

The DEE PRO role evolves as it organizes and participates in multiple R&D networks, which MNCs do not lead. The PRO increases its demand for knowledge as it seeks to develop new products for major health problems. Lacking the needed organizational capabilities, it builds networks actively, nurturing ties with foreign and local organizations, thereby integrating different knowledge bases. Existing studies highlight collaboration with one or a few MNCs in public–private partnerships for technological catch-up that facilitates innovation, usually focusing on the manufacturing process (Corredoira & McDermott, 2014; Lee & Yoon, 2015; Mazzoleni & Nelson, 2007; Parente et al., 2021). In contrast, our study shows wider diversity of knowledge sources and co-creators. As partner and founder of hundreds of different consortia, the PRO assembles diverse cross-sectoral collaborations to increase innovation capabilities. It significantly increases the number and variety of MNC partners beyond its historical practice of working with a few for production-related purposes. The diversification of MNCs includes the geographical origin and technological fields. In addition, the networks are highly diverse. They include private, public, and non-governmental organizations. The PRO uses diverse national and international inter-organizational networks to gain skills in discovery, development, and clinical research trials to overcome its historical gap in translating its generation of scientific knowledge on diseases into new technological products. As the DEE PRO increases its innovation capabilities, it also expands its ties and attracts more MNC partners for R&D purposes to join the diverse consortia of cross-sectoral organizations.

The MNC role also evolves from being an individual partner driven by corporate social responsibility (CSR), market-entry, or other commercial goals to being part of mixed R&D collaborators when the DEE PRO focuses on building innovation capabilities. Initially, the MNC is a partner for the technology transfer of existing products previously developed in the company’s home country. The MNC owns more advanced technological knowledge and product know-how and engages in relationships with DEE actors as part of its commercial strategies, i.e., to implement CSR programs in target markets to address stakeholders’ pressures and increase local reputation (Bartlett et al., 2012). The DEE goal is to manufacture the MNC’s products locally. Such a goal influences the establishment of agreements for the foreign company to provide technological knowledge for building local production capabilities (Ramani & Urias, 2018). The knowledge transfer role of the MNC collaboration with the DEE organization is also typically highlighted in the existing literature where the MNC is the primary source of knowledge for firms in DEEs (Corredoira & McDermott, 2014). However, the MNC role evolves as the PRO seeks to overcome existing gaps in R&D skills and create new-to-the-world products. Multiple MNCs with varied technological assets from different home countries join partnerships in diverse consortia that the PRO leads, promoting multi-directional knowledge flow. The DEE PRO leads the knowledge integration of the new product development. The MNC engages in NTD R&D discovery and development activities related to creating products that were not part of its existing portfolio. Instead of acting as the sole partner or sole foreign company providing knowledge, the MNC engages in mutual learning in a network where the PRO integrates multi-sourcing of knowledge from public, private, and non-governmental organizations. The MNC thus enters a new type of R&D process, which is propelled by DEEs’ social policy goals, driven by a PRO, and based on collaborative knowledge access and creation to advance the technological frontier.

The heterogeneity of collaborators means that DEEs and MNCs learn from a wide variety of knowledge sources, within and beyond their local space or one type of knowledge base, including academic centers. The existing literature on private–public networks emphasizes government organizations and firms (Anand et al., 2021; Lall, 1992; Parente et al., 2021). This study shows the PRO actively broadened the knowledge base and resources by engaging not only private firms but also academic institutions and research centers in product development partnerships over the years. The diversity of MNCs, universities, research centers, local private firms, and government organizations from heterogeneous fields and countries bring varied experience and know-how. Focusing on creating novel therapies and diagnostics, this diversity of partners is a central strategy continuously fostered and escalating. R&D networks bring together DEE universities that stand out as knowledge creation partners. Our findings support other research on new emerging technologies created in DEEs that also find a vital role for universities and research centers as strategic partners for innovation in health and bioenergy (McMahon & Thorsteinsdóttir, 2013; Perez-Aleman & Alves, 2017; Reynolds et al., 2019). In the NTD health sector, universities participate in knowledge creation in a prominent role. By joining and leading increasing collaborations with diverse cross-sectoral organizations, DEE PROs leverage varied local and foreign knowledge for building local innovation capabilities to develop health technologies. The involvement of local universities promotes and depends on building their own educational and research capabilities for innovation goals.

We also contribute a different model for building DEE innovation capabilities using strategic partnerships between PROs, MNCs, and other domestic and foreign organizations for achieving national social goals. Previous studies capture DEE firms working with foreign subsidiaries in creating new products focusing on the MNCs’ own innovation capability goal (Figueiredo et al., 2020). Other studies capture innovation capability building through GVC linkages (Lema et al., 2015a). This study demonstrates the DEE’s overall social development goals drive the strategic partnerships to create locally (and globally) relevant innovation. To gain innovation capabilities, the DEE uses a model that fosters interactions with diverse collaborators from private, public, and non-governmental sectors, including foreign MNCs. Compared to previous studies, the contribution of DEE partnerships with foreign MNCs to NTD knowledge creation is not unique nor the most important for product development. Rather, it is part of a highly diverse network of partnerships that the PRO engineers to achieve national goals. Policy priorities were also present in the case of EMBRAPA’s agricultural technological effort (Parente et al., 2021). In the health sector, social policy to address diseases affecting the poorest populations and improve health as a human right is central. The policy interactions with the knowledge networks evolution are discussed next, specifically social and innovation policies.

### Social and Innovation Policies Influence the DEE PRO’s Local and International Network Evolution

This study shows that DEE social and innovation policies influence the PROs’ pursuit of local and international connectivity by creating and participating in multi-directional distributed (local and global) knowledge generation networks. The leveraging of cross-border sources from diverse public and plural partnerships nurtures a local innovation process. DEE social and innovation policies drive the growing number and variety of knowledge networks. The DEE social policy creates demand for knowledge generation following a government shift to provide free and universal access to healthcare. The DEE innovation policy promotes knowledge creation in a context of minimal technological development and dominance of imports from foreign MNCs. While the policies promote knowledge seeking and generation to address major health problems, the PRO organizes and creates collective inventions typically captured in studies of advanced economies. Collective invention where knowledge sharing does not occur through the conventional MNC R&D lab is a feature of current innovation organizing in North America and Europe (Powell & Giannella, 2010). The DEE PRO leverages the new forms of organizing collective invention that is distributed across diverse organizations locally and globally, which provide opportunities for knowledge sharing and creation in ways that bring in, but do not depend on, MNCs.

The DEE social policy compels the government to increase both production and innovation capabilities. The goal of increasing production capabilities influenced the emergence of ties between the PRO and MNC focused on technology transfer to manufacturing locally existing products. At the same time, the social policy influenced innovation policy, as the DEE established health sector innovation as a strategic priority, which highlighted how addressing the NTD challenge requires advancing the global technological frontier. Integrating national health and innovation goals generates policies toward patenting, R&D incentives for tests and diagnostics, investments in R&D, strengthening of public sector labs and universities, and clinical studies. There is also funding for prototyping. Healthcare innovation focused on NTDs in the 2000s and 2010s, initiating an NTD R&D Program. While health policies create demand for knowledge, the innovation policy and the PROs strategy aim to overcome a disconnect with healthcare needs. Innovation policy moves beyond a focus on science and technology to give attention to major health challenges; in turn, the investments in health innovation advance and promote DEE innovation capabilities. In the case of Brazil, the MoH fostered research in health, R&D, and clinical research, providing funding for innovation projects accounting for 1.5% of its budget (Victora et al., 2011). Innovation laws also favored the health sector (Shadlen & Fonseca, 2013). Our study is the first to show the evolution in knowledge networks that emerge following the national policy goal to increase innovation to improve and increase access to healthcare, particularly for the poorer population.

The proactive effort to overcome gaps and weaknesses in innovation capabilities in an integrated manner with a strong social policy to expand healthcare to poorer groups presents a new approach to understanding innovation in DEEs. Existing studies capture the policies to build production capabilities to integrate suppliers in MNC-driven supply chains and for exports (De Marchi & Alford, 2021; Manning & Richter, 2022; Pietrobelli, Rabellotti, & Van Assche, 2021). Other studies reveal policies that promote local production to replace and reduce dependence on imports of pharmaceuticals (Horner, 2022; Kaplan & Laing, 2005; Mackintosh, Banda, Tibandebage, & Wamae, 2015). Research has also focused on the pre-requisites for fostering DEEs’ new industrial policies’ ability to expand the global technological frontier in strategic industries but disconnected from social policy goals (Amsden, 2001; Fuentes & Pipkin, 2019). Some studies, however, show a strategy that connects innovation to social policy and industrial policy (McMahon & Thorsteinsdóttir, 2013; Perez-Aleman & Alves, 2017). This study advances this literature by showing how government policy interacts with the evolution of knowledge networks. Networks evolve as government social and innovation policies evolve, influencing the types of linkages established with local and foreign organizations, including MNCs.

As innovation in DEEs plays an increasingly important role, this study captures the DEE government's strategy to foster learning conditions. The role of government in promoting learning to build DEE capabilities is well established in the literature (Amsden, 2001; Cimoli, Dosi, & Stiglitz, 2009; Perez-Aleman and Alves, 2017; Pipkin & Fuentes, 2017; Rodrik, 2007; Sabel, 1994). Previous studies mostly focused on production capability building, leaving partially unaddressed the need to understand how government policies in DEE can also build innovation capabilities that advance the technological frontier, not just production capabilities (Fuentes & Pipkin, 2019). That is also the case for work highlighting that DEE governments foster learning connected to local problems in agriculture, manufacturing, health, information technology, and finance (Lema, Iizuka, & Walz, 2015; Mazzucato, 2018; Parente et al., 2021; Shadlen & Fonseca, 2013; Stiglitz et al., 2013). This study adds to growing recent research on learning to build innovation capabilities in DEEs by revealing how local problems drive demand for policies to address them and how the social policies that originate push the creation of new technological knowledge (Perez-Aleman & Alves 2017). We show that DEEs leverage a two-way interaction between social and innovation policies to drive PRO-engineered collaboration networks with local and global reach. How the DEE organizes its networks matters as innovation depends on distributed and cross-sectoral knowledge.

The DEE use of knowledge networks for collective invention enables DEEs to bring together complementary know-how to generate the frontier technological innovation required to address urgent social needs. This approach integrates the current understanding that DEE governments steer industrial policies internally to generate market demand and production capabilities for products based on existing foreign knowledge to satisfy social welfare needs (Horner, 2022; Shadlen & da Fonseca, 2013; Stiglitz et al., 2013). The combination of social and innovation policies pushing the long-term strategic development of complex research-intensive networks further enriches the national industrial system beyond production and trade, creating innovation capabilities that bring national PROs to the fore of global R&D activities, re-shaping the production and innovation landscape traditionally dominated by MNCs. We show that connecting locally and globally across diverse cross-sectoral organizations becomes vital for NTD innovation capability building in the context of socially driven innovation. International NGOs and universities are as important as connecting with foreign MNCs. This is consistent with recent literature showing frontier innovation in DEEs where local organizations develop new-to-the-world technology (Lema & Lema, 2012; McMahon & Thorsteinsdóttir, 2013; Perez-Aleman & Alves, 2017). They build new capabilities through participation in local and international networks with a plurality of knowledge pipelines from diverse plural actors. In NTD innovation, DEE actors assume leadership and agency, rather than being subordinate contributors, actively fostering the creation of strategic networks for knowledge creation. Existing literature examines how DEE governments directly contribute to the innovation capabilities of domestic firms, primarily through PROs and universities, with firms remaining the central actors in the innovation dynamics (Laursen & Santangelo, 2017; Malerba & Nelson, 2011; Parente et al., 2021; Pietrobelli & Rabellotti 2011). We expand this literature by emphasizing how DEE PROs access local and foreign sources of know-how by establishing linkages with diverse public, private, and non-governmental organizations to create new knowledge for addressing major domestic health challenges. They are driven by the social goals of creating affordable, accessible technologies for improving healthcare for the disadvantaged. These networks of diverse partners represent alternatives for knowledge building nationally and across borders.

The DEE PRO promotes innovation capabilities internally and externally to produce local solutions to national policy priorities. However, the diverse networks that emerge engage in new international collaborations where they access and contribute complex knowledge for global health. Locally developed knowledge feeds the exploration of foreign knowledge to generate new solutions of relevance for global health and SDGs. The existing literature explains the MNC-driven global knowledge creation dynamics (Bathelt & Li, 2020; Cano-Kollmann et al., 2016; Lema et al., 2015b). This study adds a different strategy focused on NTD innovation that DEE public and civil society actors drive, which often follows south–south knowledge flows. For example, the connections between Brazil and other DEE governments to address NTD health problems indicates leadership from the south in global knowledge creation networks following a social development agenda.

### Inclusive Innovation and Local–Global Partnerships to Address Sustainable Development Goals

This study contributes to the growing international business literature addressing social and environmental challenges by analyzing a DEE strategy to address health improvement. The SDGs are directly relevant to international business, and diseases that afflict the poorest are central concerns of SDG #3 *Ensure Healthy Lives and Promote Well-Being*. Existing research highlights the contribution of MNCs to the creation of positive sustainability outcomes, either in their global operations or through the products they commercialize for low-income populations (Buckley, Doh, & Benischke, 2017; Kolk, Kourula, & Pisani, 2017; Prahalad, 2004). Studies note MNCs and their subsidiaries need to build capabilities to generate SDG-related knowledge to address social and environmental issues and act responsibly (Montiel et al., 2021). Increased connectivity through new ties across different sectors is crucial to facilitate the creation of such capabilities (van Zanten & van Tulder, 2018). MNCs increasingly partner with various public actors to develop innovation that addresses social and environmental issues (Lundan & Cantwell, 2020). MNC’s connectivity to local knowledge across multiple locations is crucial for its innovation capability (Cano-Kollmann et al., 2016). MNCs benefit from accessing multiple knowledge sources and entering mutual learning dynamics with DEE organizations (Figueiredo et al., 2020; Herrigel et al., 2013; Lema et al., 2015b). We show that DEE social and innovation policies and the PRO influence the MNCs’ ability to innovate for sustainability and positive social impact, as it depends on partnering with DEE organizations in knowledge creation collaborations. Innovating products to combat diseases affecting the poor is not done by MNC R&D labs alone but as part of cross-sectoral collaborations that DEEs actively advance for social policy goals promoting inclusive innovation. The MNC learns from the DEE PRO and government when addressing grand challenges such as SDGs.

This study also expands the understanding of forms of organizing innovation to address global health challenges central to the SDGs from the DEE perspective. Existing literature examines the organizing of global health R&D for DEE challenges through Product Development Partnerships (PDPs), focusing on organizations from advanced economies (Chataway, Hanlin, Mugwagwa, & Muraguri, 2010; Pereira et al., 2020). Global R&D PDPs with diverse cross-sector organizations develop NTD solutions given the lack of commercial interest in DEE health issues (Muñoz et al., 2015). Global health R&D PDPs accounted for 75% of NTD drug development projects worldwide (Chataway et al., 2010). However, existing research on global PDPs does not address the role that DEE organizations play in their development and governance, especially PROs. Public–private partnerships to develop an AIDS vaccine and a malaria vaccine involve interactions between government and firms to develop technologies for poorer groups. However, studies highlight North American and European actors such as foundations and financing sources (Chataway et al., 2010; Pereira et al., 2020). This study adds a DEE perspective of the active role of DEE PROs and their strategy to build innovation capabilities by leveraging partnerships. In this case, the PRO creates R&D partnerships with diverse organizations, including MNCs, to tackle local health challenges with global ramifications. The PRO also participates as a leader of global R&D PDPs. Moreover, the national social and innovation policies support the implementation of global R&D initiatives.

We show that DEE PROs assume leadership in organizing PDPs to build up NTD innovation capabilities. The PRO simultaneously assembles, leads, stimulates, and expands across geographies a multiplicity of PDPs targeting different NTDs. Multiple PDPs emerge within a broader network of strategic partnerships that fit the DEE PRO mission to innovate NTD products. The cross-sector collaborations identified in this study are strategic networks formed locally and internationally that concentrate on R&D, which differ from the production supply chains governed by MNCs. R&D collaborations between diverse organizations are a feature of networks of biotech innovators in advanced economies (Powell & Grodal, 2005). However, they are not associated with innovation in developing economies, as the literature highlights sources of knowledge from foreign MNCs and local firm interactions. Nevertheless, this study indicates that knowledge sourcing is not limited to MNC channels, although it includes them as partners in cross-sectoral partnerships led by PRO. Partnerships with MNC alone do not characterize the pathway for NTD innovation. By drawing on diverse local and foreign collaborators, DEE PROs and other organizations engage with more complex know-how, creating an alternative path in a global health structure in which knowledge production from advanced countries traditionally went in a unidirectional flow from North-South. Instead, DEE organizations use broad-based cross-sector collaborations to become knowledge makers for NTD innovation through ties between government, MNCs, and other private and plural organizations, including their local universities.

Social policies benefitting lower-income groups also coincide with NTD innovation capability building. The existing literature notes the need to expand the understanding of new models of inclusive innovation to tackle societal challenges (George, McGahan, & Prabhu, 2012; Pereira et al., 2020). The urgency to address social challenges requires new forms of organizing innovation that overcome the lack of MNCs’ investments and create access to the sources of knowledge required to develop products for disadvantaged groups (George et al., 2012). The PRO facilitates the creation of consortia and R&D networks involving MNCs in NTD innovation. MNCs contribute toward an R&D process that has the primary goal of addressing social development goals. DEE PROs bring in MNCs as partners while implementing a social policy that fosters inclusive innovation processes. National social policies in a developing country drive NTD innovation efforts that foster interactions between SDGs and MNCs. In this study, the Brazilian government of the 1990s and 2000s saw improved healthcare as the strategic driver of innovation. NTD initiatives accompanied other social initiatives to expand human capabilities, such as anti-poverty programs, cash transfers to the poor, and child education (Gibson, 2017). Importantly, these efforts do not remain concentrated at the national level, as networks with a variety of local organizations and universities distribute knowledge and capability building to different regions in the country.

## Conclusions

This study advances international business research by increasing understanding of how DEEs can construct knowledge networks that create innovation capabilities related to improving the health of the poor. Cross-border collaborations bring dispersed and diverse knowledge for collective invention to address major health challenges. We uncover an evolution of these knowledge networks where both the government and MNCs' roles change in interaction with social and innovation policies. The PRO actively builds capabilities by establishing networks of local and international actors and assembling diverse private, public, and NGO organizations, including research centers, universities, and public agencies, along with local and foreign firms. We show a dynamic in which the more the DEE PRO aims to innovate, the more diversified the knowledge sources become. For its part, the MNC role shifts away from bilateral partnerships oriented to production and technology transfer of existing products toward joining multi-organizational networks of diverse types of organizations. DEE knowledge-seeking to produce existing products activates bilateral ties with one MNC. However, the DEE PRO’s knowledge sourcing and generation for innovation assemble many MNCs in a collective effort with varied local and foreign organizations, including universities. R&D projects focus on technological products the MNC has not developed and neglected in the past. The DEE PRO activates diverse local and international networks in multi-directional knowledge sharing. DEEs move into new knowledge creation and all aspects of new product development. DEE PROs strategically use multi-level local and global collaborations to access, leverage and generate new knowledge, where MNCs join many DEE partners in diverse networks that provide conditions for DEE learning and innovation capability-building goals.

National policies drive the PRO to source and create knowledge for innovation connected to the goal of improving the health of disadvantaged groups. The PRO leads the implementation of government social policies to increase access to and improve healthcare. Social and innovation policies influence the characteristics and evolution of knowledge networks. Social goals to improve the health of the poor create demand for inventions for NTDs. They also influence innovation policies that place health challenges as strategic for social and economic development. The integration of social and innovation policies promotes investments in R&D for health. National policies to address local healthcare propel innovation of local and global relevance. Local initiatives escalate into global NTD efforts. MNCs engage with health challenges related to the SDGs as part of the consortia that the DEE promotes, where PROs lead cross-sectoral collaborations. National government social goals influence the way MNCs interact and share knowledge in R&D alliances while contributing to the health dimension of the SDGs. Given the MNCs' historical lack of investment in NTDs, their contribution to improving local and global health depends on DEE national policies that create demand and innovation capabilities for SDG health solutions, which attract and foster MNC interactions in cross-sectoral networks analyzed in this study.

These novel findings on forms of organizing knowledge networks for innovation are also relevant for current efforts to combat NTDs, confront global pandemics, mitigate the effects of climate change, promote sustainability, and advance the SDGs. We show how DEE innovation capabilities to address major social challenges contribute to SDG health solutions. Moreover, as the COVID-19 outbreak has a devastating impact worldwide, this study suggests that innovation capabilities to address local and global issues need knowledge sharing through international channels beyond MNCs alone to generate common solutions to global challenges that improve the lives of the poor and social conditions. The cross-sectoral partnerships that characterize the DEE PRO strategy in NTDs have relevance for other diseases such as COVID-19 (Fu, Buckley, Sanches-Ancochea, & Hassan, 2021). Some studies show these structures include partnerships between NIH and Moderna, or the Biomedical Advanced Research and Development Authority, focusing on northern countries (Pereira et al., 2020). However, access to new COVID vaccines invented in Western countries did not reach many DEE countries. In March 2022, 73%of EU residents were vaccinated compared to 13% in Africa (Barnes, 2022). Left out, given highly unequal access to COVID vaccines, African health ministries are making plans to foster skills to produce vaccines with South African biotech companies. This study shows the knowledge networks that could facilitate capability building in other DEEs. Our work advances the understanding of these public–private partnerships in DEE contexts that are central to the SDGs.

This study also offers relevant policy implications for DEEs’ specific efforts to address major health and SDG challenges. There is a need for policies that support science and R&D investments in DEE PROs and national universities connected to major social challenges. Policies enabling the international connectivity of such actors are essential. Knowledge creation for addressing SDG challenges such as health and NTDs depends on facilitating collective invention with diverse communities of organizations in which DEE actors have leadership. The role of international organizations, such as the United Nations, and global platforms for the governance of R&D collaboration, such as DNDI, will play a key role in addressing those issues and enabling DEEs’ efforts in innovation capability building (Ambos & Tatarinov, 2022; Hoekman & Nelson, 2018; Van Assche & Brandl, 2021; Sachs et al., 2019). Integrating innovation and health policies in DEEs’ development agenda is crucial for advancing SDG solutions. Future research could focus on how DEEs’ national policy instances and implementation strategies connect to and interact with global efforts to achieve the SDGs.

While this study focuses on the health sector, future studies could explore the interactions and networks of DEE PROs, MNCs, and diverse organizations in relation to current priorities outlined in the SDGs, climate action, and other global health challenges. Other social sectors targeted by the SDG goals could be explored to expand understanding of the interactions between innovation, local and global firms, and national policies arising to meet the SDGs. Future studies could explore the DEE actors’ role in pursuing innovation in other social sectors and how it shapes MNCs' strategies and partnerships. In addition, the strategy of using diverse partnerships could be expanded to analyze MNCs' engagement with other sectors as they seek to increase social impact. We conducted this research prior to the COVID-19 pandemic. In this respect, the COVID-19 pandemic brought to light the importance of inventing and producing vaccines, tests, and drugs that are affordable and accessible to DEEs. The solutions that developing countries innovate also have global implications and relevance. Two-way interactions with developing countries contribute to the local and global solutions. More studies are needed to understand the post-pandemic effects on the DEE innovation capability-building strategies in health. As well, future research can build on the novel insights of this study to understand both the DEE role and interactions with MNCs in innovation targeting SDGs that require science-intensive solutions.
